# Bidirectional cytokine-microRNA control: A novel immunoregulatory framework in leishmaniasis

**DOI:** 10.1371/journal.ppat.1010696

**Published:** 2022-08-04

**Authors:** Abdollah Jafarzadeh, Maryam Nemati, Najmeh Aminizadeh, Neelam Bodhale, Arup Sarkar, Sara Jafarzadeh, Iraj Sharifi, Bhaskar Saha

**Affiliations:** 1 Department of Immunology, School of Medicine, Kerman University of Medical Sciences, Kerman, Iran; 2 Molecular Medicine Research Center, Research Institute of Basic Medical Sciences, Rafsanjan University of Medical Sciences, Rafsanjan, Iran; 3 Immunology of Infectious Diseases Research Center, Research Institute of Basic Medical Sciences, Rafsanjan University of Medical Sciences, Rafsanjan, Iran; 4 Department of Haematology and Laboratory Sciences, School of Para-Medicine, Kerman University of Medical Sciences, Kerman, Iran; 5 Department of Histology, School of Medicine, Islamic Azad University Branch of Kerman, Kerman; 6 National Centre For Cell Science, Pune, India; 7 Trident Academy of Creative Technology, Bhubaneswar, Odisha, India; 8 Student Research Committee, School of Medicine, Kerman University of Medical Sciences, Iran; 9 Leishmaniasis Research Center, Kerman University of Medical Sciences, Kerman, Iran; Institut Pasteur, FRANCE

## Abstract

As effector innate immune cells and as a host to the protozoan parasite *Leishmania*, macrophages play a dual role in antileishmanial immunoregulation. The 2 key players in this immunoregulation are the macrophage-expressed microRNAs (miRNAs) and the macrophage-secreted cytokines. miRNAs, as small noncoding RNAs, play vital roles in macrophage functions including cytokines and chemokines production. In the reverse direction, *Leishmania*-regulated cytokines alter miRNAs expression to regulate the antileishmanial functions of macrophages. The miRNA patterns vary with the time and stage of infection. The cytokine-regulated macrophage miRNAs not only help parasite elimination or persistence but also regulate cytokine production from macrophages. Based on these observations, we propose a novel immunoregulatory framework as a scientific rationale for antileishmanial therapy.

## 1. Introduction

**Fig 1:**
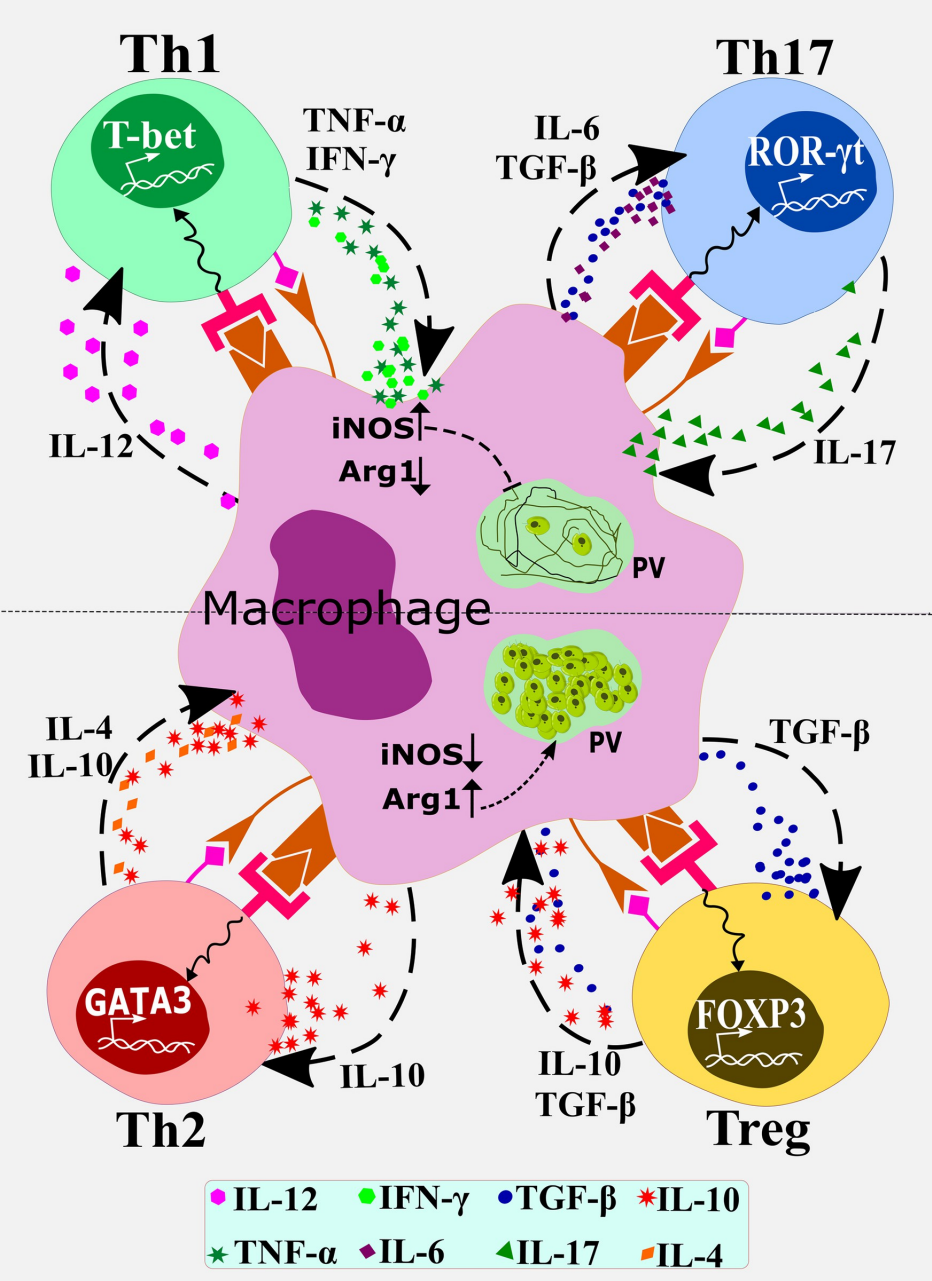
A basic macrophage-T cell subset differentiation scheme that counteractively regulates the alternative outcomes of *Leishmania* infection. Macrophages are the host cells for the protozoan parasite *Leishmania* (PV with amastigotes are shown in green). The top half (above the broken line) shows the condition when Th1 and Th17 cells are differentiated in presence of the respective signature transcription factors T-bet and ROR-γt. These cells activate macrophages to induce iNOS but to reduce arginase (Arg1). iNOS catalyzes the formation of nitric oxide that kills the amastigotes within macrophages. The bottom half shows the differentiation of Th2 ad Treg cells in presence of the respective signature transcription factors GATA-3 and FOXP3. IL-4 and IL-10 secreted by these cells deactivate macrophages to reduce iNOS, but increase Arg1, expression resulting in parasite growth in macrophages. miRs target these macrophage–T cell interactions to manipulate the outcomes of *Leishmania* infection. iNOS, inducible nitric oxide synthase; PV, parasitophorous vacuole.

Tissue macrophages are differentiated from the bone marrow–derived monocytes that are recruited to the tissues [1]. During chronic infections, progenitor cells are mobilized from bone marrow, recruited at the site of infection, and the macrophages can be differentiated in situ. Macrophages play dual roles in many infectious diseases: playing host to an intracellular pathogen such as *Leishmania* or acting as an executioner eliminating the pathogen by eliciting antileishmanial immune effectors such as cytokines [1,2]. Thus, macrophages can be polarized to host-protective (M1 macrophages) or proparasitic phenotype (M2 macrophages) [3]. Macrophages are the major producers of IL-1β, IL-6, IL-12, IL-10, and IL-23 [4]. IL-12 contributes to Th1 cell polarization, while IL-1β, IL-6, and IL-23 trigger and maintain Th17 cell polarization [5]. Although Th1 and Th17 cell subsets play complementary roles in protecting against *Leishmania donovani* and *Leishmania infantum* infections, there are ambiguities regarding the role of Th17 cells in *L*. *major* infection [6,7]. IFN-γ and TNF-α from Th1 cells activate macrophages to induce NO and ROS eliminating *Leishmania*, while IL-4, IL-10, and TGF-β from Th2 and Treg cells inhibit the leishmanicidal functions of macrophages [6,7]. Macrophage-derived IL-10 and TGF-β exert preventive effects on the M1 macrophages and Th1 cells while promoting Th2/Treg cell differentiation [8,9]. A basic macrophage—T-cell network is shown in Fig 1.

**Fig 2:**
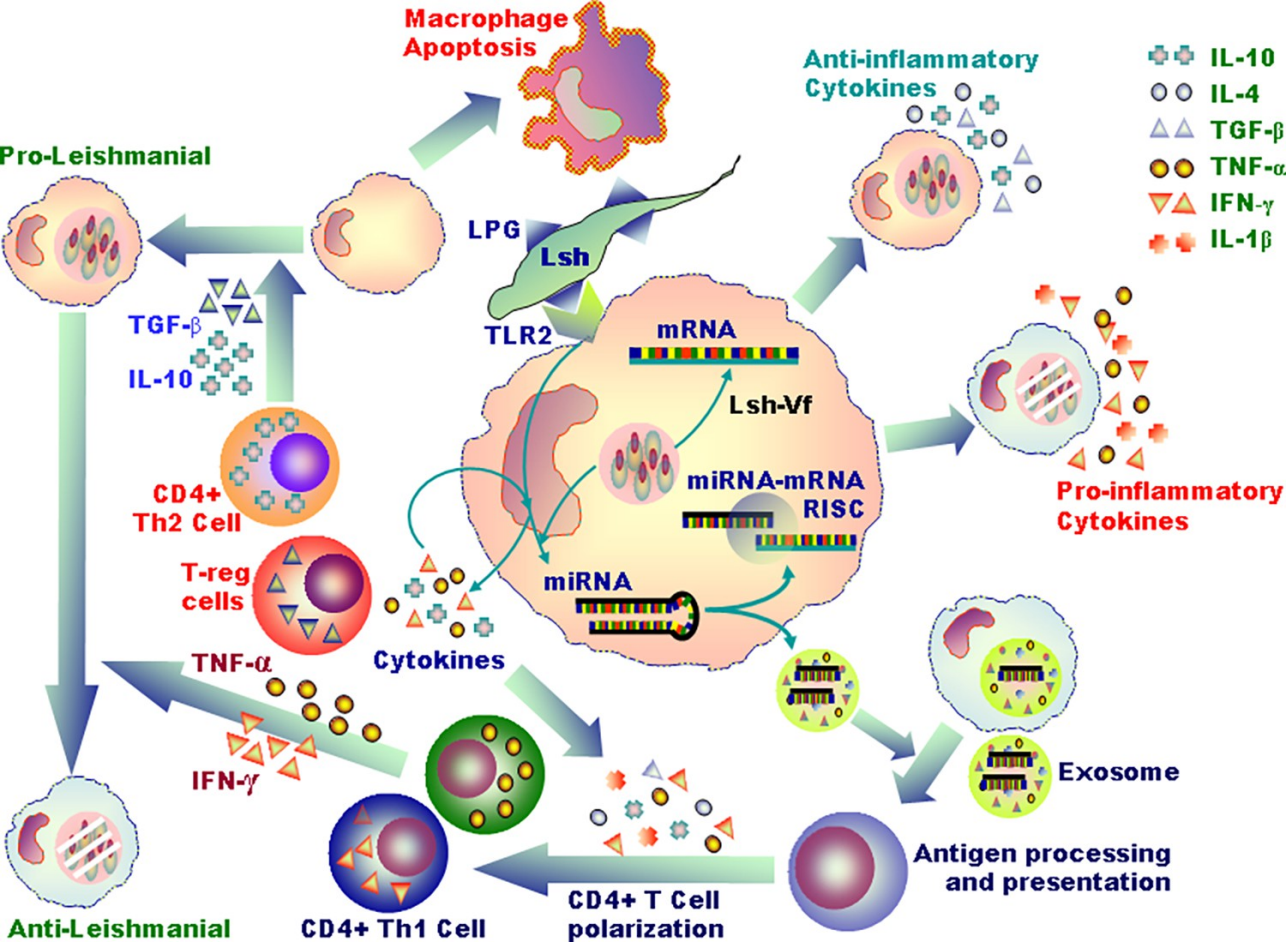
*Leishmania*-expressed ligands such as LPG interact with corresponding receptors (e.g., TLR2 for LPG) on macrophages and signal altered miRNA expression (at the centre of the figure). As shown clockwise surrounding the central macrophage, these miRNAs affect macrophage production of proinflammatory and anti-inflammatory cytokines, antigen processing and presentation of macrophages, CD4^+^ T cell polarization into opposing effector subsets, antileishmanial or proleishmanial effects, and macrophage apoptosis or survival. Proleishmanial to antileishmanial functions are induced by the Th1 cell secreted IFN-γ and TNF-α. A balance between these counteractive functions regulated by miRNAs, which can be altered by *Leishmania*, determines the outcome of the infection. The *Leishmania*-infected macrophages secrete exosomes filled with different antigens and miRs. The exosomes can be taken up by the neighboring uninfected macrophages, wherein these miRs can execute their functions. The key to the cytokines are shown on the upper right corner of the figure. LPG, lipophosphoglycan; miRNA, microRNA.

In this intriguing battle between the host and the parasite, *Leishmania* the parasite enters the host macrophages and subverts its defense mechanisms—phagosomal maturation, MHC class-I and -II-mediated antigen presentation, inflammatory cytokine productions, activation of leishmanicidal mechanisms such as generation of reactive oxygen species (ROS), and nitric oxide (NO)—and multiply within phagolysosomes [10–12]. On the other hand, macrophages marshal an intense but diverse immune attack on the parasite to eliminate it. As these 2 counter-forces face off, microRNAs (miRNAs) regulate the outcome of the duel (Fig 2).

miRNAs are 20 to 22 nucleotides long noncoding RNAs regulating gene expression by target mRNA degradation or translational repression [13,14] and potentiate or inhibit macrophage activities against infectious agents [13]. We propose that *Leishmania*-regulated aberrant miRNA expression in macrophages affects the production of leishmanicidal cytokines, whereas macrophage-deactivating cytokines regulate miRNAs to support the parasite. Finally, we invoke an immunoregulatory framework as a scientific rationale for designing an antileishmanial therapy.

## 2. The biogenesis of microRNAs

**Fig 3:**
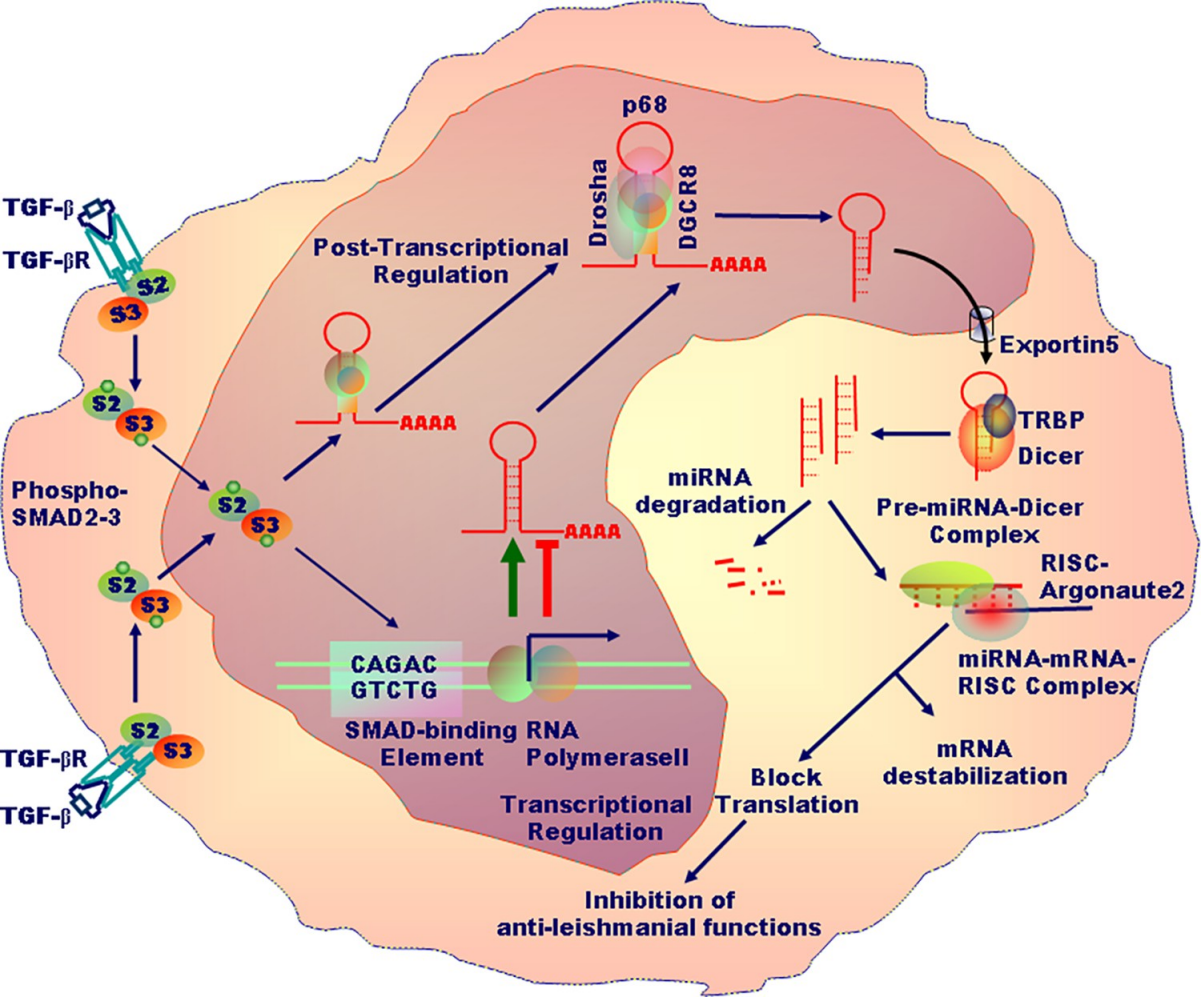
TGF-β induced by *Leishmania* infection binds to TGF-β receptor causing its phosphorylation and subsequent SMAD2/SMAD3-dependent transcriptional and posttranscriptional regulation of miRNA expression in macrophages. These miRNAs make complexes with the target mRNAs—one miRNA binding to many mRNAs—to destabilize or suppress mRNAs translation. Depending on the miRNAs up-regulated or down-regulated, proleishmanial or antileishmanial effects are observed. miRNA, microRNA.

Within the nucleus, RNA polymerase II transcribes the DNA to primary miRNA (pri-miRNA; >1 kb) with a local stem-loop structure, wherein the mature miRNA is embedded [15,16]. The pri-miRNA is then cropped by the RNAse III Drosha and its key cofactor, DGCR8 (DiGeorge Syndrome Critical Region 8) to create a small hairpin-shaped miRNA precursor (pre-miRNA; approximately 65 nucleotides). Once exported to the cytoplasm, pre-miRNA is further pruned by an RNAse III Dicer to generate the miRNA duplex, which is next loaded onto Ago2, an Argonaut protein, to form the effector complex called RISC (RNA-induced silencing complex) [16]. Within the RISC, Ago2 guides the binding of the nucleotides 2 to 8 of the 5′ end of miRNAs to a complementary region of 6 to 7 nucleotides placed in the 3′-untranslated region (UTR) of target mRNAs [15,16] (Fig 3).

## 3. *Leishmania* regulates microRNAs expression in macrophages

*L*. *major*, *L*. *donovani*, *L*. *amazonensis*, *or L*. *infantum* infect macrophages—of both mice and humans—altering the expression of numerous miRNAs [17–21] that inhibit the leishmanicidal functions of macrophages [22]. These miRNAs target various biological processes such as macrophage polarization, NO production, apoptosis and autophagy, cytokine and chemokine production, phagosome maturation, drug response, and T-cell polarization [23,24] (Fig 1).

The *L*. *major* and *L*. *donovani*-infected human macrophages and dendritic cells (DCs) exhibit different patterns of miRNA expression [21]. Parasite strain, isolate, load, and virulence factors may affect the expression of macrophage miRNAs. For example, in the *L*. *infantum-*infected dogs PBMCs, miR-194 and miR-371 expression show a positive association, but miR-150 expression shows a negative relationship, with the parasite load [25]. Among the same species of *Leishmania*, different strains can exhibit differential effects on miRNA expression [21]. A more infective *L*. *infantum* strain causes higher miR-155 expression in macrophages than a less infective strain [20]. *L*. *donovani* isolates from visceral leishmaniasis (VL) and post-kala-azar dermal leishmaniasis (PKDL) patients differentially influenced the expression of miRNAs in the THP-1-derived macrophages. Differentially expressed miRNAs influence various biological processes such as PI3K signaling, cell cycle regulation, immunomodulation, apoptosis inhibition, and cytokine production [26].

In humans, most miRNAs were overexpressed in *L*. *donovani*-infected, but not as much in *L*. *major*-infected, macrophages and DCs [21]. Although up-regulation of many miRNAs in *L*. *donovani*-infected macrophages was reported, miRNA down-regulation was attributed to the enhanced stability of c-Myc, a transcription factor that binds to the miRNA-related promoters and influences deacetylation to repress miRNA biosynthesis but promote parasite survival; c-Myc blockade reduced amastigote survival [27,28]. Wild-type and arginase-deficient *L*. *amazonensis* differentially influence miRNA expression in mouse macrophages suggesting a role for *Leishmania*-expressed arginase in modulating the macrophage miRNA expression [19].

**Fig 4:**
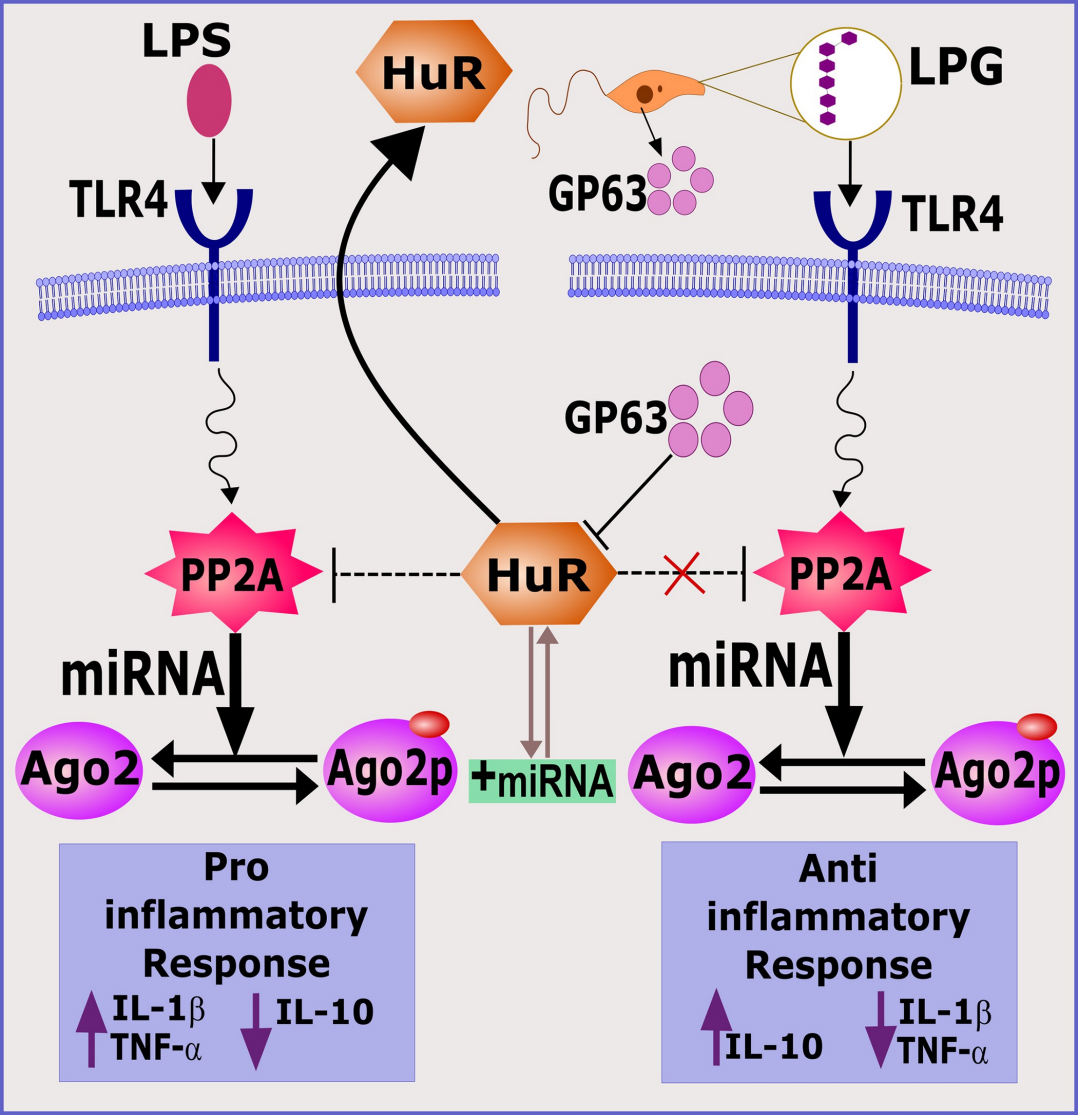
HuR and Phosphatases reciprocally regulate proinflammatory and anti-inflammatory cytokines. While Protein Phosphatase 2A (PP2A) increases miRNA activity, HuR overexpression down-regulates PP2A activation, and thereby miRNA expression, leading to increased proinflammatory cytokines. *Leishmania*-derived protease Gp-63 degrades HuR making active PP2A available for enhancing anti-inflammatory cytokines. On the other hand, *Leishmania*-derived LPG may activate TLRs to regulate PP2A activation that through regulation of argonaute proteins modulates cytokine response. LPG, lipophosphoglycan; miRNA, microRNA.

Antimony-resistant (R-LD) and antimony-sensitive *L*. *donovani* (S-LD) selectively influence miRNAs in macrophages leading to discernible cytokine responses. *R-LD* and *S-LD* up-regulate protein phosphatase 2A (PP2A) but down-regulate Hu-antigen R (HuR) at different magnitudes. PP2A increases miRNA activity while HuR antagonizes it, leading to anti-inflammatory or proinflammatory cytokine responses, respectively [17]. Ago2 phosphorylation impairs its ability to bind target mRNAs but *Leishmania*-induced PP2A-mediated dephosphorylation reactivates it [17]. The binding of dephosphorylated Ago2 with miRNAs suppresses the expression of miRNA-associated proinflammatory cytokines in infected macrophages [17,29]. As HuR inhibits PP2A expression, HuR overexpression induces a significant proinflammatory macrophage response reducing *L*. *donovani* infection [29] (Fig 4).

*Leishmania amazonensis-*expressed phosphatidylserine (PS) on the outer side of the cytoplasmic membrane is recognized by macrophage-expressed molecules and may cause silent invasion of macrophages without activation and increase parasite proliferation by IL-10 and TGF-β induction [30,31]. In an *L*. *donovani* infection model, macrophages dynamically regulate more than 900 miRs, of which miR-6540 may target PS and influence the parasite’s fate within macrophages [22]. However, the exact mechanism of PS-miRNA-6540 interaction awaits elucidation. Once inside, the parasite proliferates causing a hypoxic condition that induces hypoxia-inducible factor-1α (HIF-1α) expression potentiating macrophage-mediated immunity against *L*. *amazonensis* and *L*. *major* while attenuating the responses against *L*. *donovani* [8,32]. miR-3620 and miR-6385 are highly elevated in *L*. *donovani* infected macrophages, which probably down-regulate hypoxia-induced genes [22]. miR-3620 up-regulation controls iron homeostasis-related genes to ensure maximum iron availability to the parasite [22]. In *L*. *donovani*-infected macrophages, miR-210 is up-regulated in a HIF-1α-dependent manner and targets NF-κB p50 and TNF-α receptor mRNAs, limiting the production of proinflammatory cytokines while enhancing IL-10, aiding parasite survival. HIF-1α and miR-210 silencing promotes TNF-α and IL-12, but reduces IL-10, expression in these macrophages [33].

## 4. Macrophage-related microRNAs influence the macrophages polarization

Proinflammatory macrophages or M1 cells express proinflammatory genes such as IL-1β, IL-12, IFN-γ, and TNF-α in NF-κB-, STAT1-, interferon regulatory factor 1 (IRF-1)-, activator protein 1 (AP-1)-dependent manner [34,35]. M1 macrophages up-regulate NOS2 but down-regulate Arginase 1 (ARG1), limiting the parasite infection [36] while the reverse is observed in M2 macrophages [18]. Th2 cytokines such as IL-4, IL-10, IL-13, TGF-β, and M-CSF promote M2 polarization with the transcription factors STAT6, CCAAT/enhancer-binding protein beta (c/EBPβ), and surface molecules chitinase 3 like-3 (YM-1) and resistin-like molecule alpha (FIZZ1) as markers [34,35].

While miR-26a, miR-26b, miR-125a, miR-125b, miR-155, miR-181, and miR-720 contribute to the induction of M1 macrophages but suppressing M2 phenotype [13,37], whereas miR-146a, miR-146b, miR-125a, let-7c, and miR-181a contribute to the induction of M2 macrophages but suppression of M1 phenotype [13,37]. The miR-125a and miR-125b can up-regulate the M1 macrophage markers, while repressing M2 macrophage polarization by targeting interferon regulatory factor 4 (IRF4) [38]. However, Banerjee and colleagues indicated that Kruppel-like factor 13 (KLF13), a transcriptional factor that play a key role in M1 macrophage activation, is targeted by miR-125a-5p, which suppresses M1 macrophage activity, while increasing M2 macrophage response [39]. It is thus possible that miR-125 may both suppress and activate macrophages depending on the existing state of cellular activation and availability of a co-factor. Such a duality has also been reported for miR155, as discussed elsewhere in this review. miR-720 inhibits M2 polarization by reducing GATA3 expression, an M2-related transcription factor [40]. The importance of these M1- and M2-inducing miRNAs in leishmaniasis remains less understood. In *L*. *donovani*-infected BALB/c monocyte-derived macrophages (MDMs) and in spleen/liver, up-regulation of M2 macrophage-related miRNAs (miR146a-5p, miR-181a-5p, and miR-125a-5p) and down-regulation of an M1 macrophage-related miRNA (miR26a-5p) were observed [36]. miR-146a-5p down-regulates iNOS, but promotes Arg1, expression during *L*. *donovani* infection. miR-146a-5p inhibition reduced the expression of M2 markers and parasite burden but enhanced the expression of M1 markers (iNOS and IL-12) in the spleen and liver of these *L*. *donovani*-infected mice. The silencing of the bromodomain-containing protein 4 (BRD4), an epigenetic and transcriptional regulator, abrogated miR-146a-induced M2 polarization suggesting that BRD4-dependent super-enhancer regulated miR-146a expression in *L*. *donovani* infection [36]. By contrast, miR-146a is up-regulated in *L*. *major*-infected human macrophages and aids in killing of the parasite by targeting the SMAD4 in the TGF-β signaling cascade [41]. Although these discrepancies remain unsolved till date, it is plausible that the *L*. *donovani* and *L*. *major* express different LPGs and thereby interacts with different heterodimers of TLR2 to trigger opposite outcomes of the infection. Similar paradox exists in miR-155 functions as well. On the one hand, miR-155 promotes M1 polarization while preventing M2 polarization and facilitates *Leishmania* elimination [3,37,42]; on the other hand, miR-155 promotes *Leishmania* persistence via inhibition of macrophage apoptosis [43]. Thus, a balanced miR-155 expression within macrophages needs to maintain M1 macrophage polarization without repressing apoptosis.

## 5. Macrophage-related microRNAs influence the NOS production

In macrophages, the up-regulation of NOS2 and NO production during the initial steps of infection exerts robust leishmanicidal activity [7,44]. Th1-related cytokines including IL-2, IFN-γ, and TNF-α induced NO production that reduced the infectivity, whereas Th2- and Treg-related cytokines such as IL-4, IL-10, and TGF-β suppress NOS2 expression promoting infectivity [7,44]. In *L*. *amazonensis*-infected macrophages, down-regulation of miR-30e-5p, miR-181c-5p, and miR-302d-3p leads to the up-regulation of NOS2 expression, which could reduce infectivity [18]. The expression of miR-294-3p and miR-721 is increased in *L*. *amazonensis-*infected macrophages up to 48 hours following infection, targeting NOS2 mRNA; reduced NOS2 expression and NO production support the *L*. *amazonensis* infectivity. miR-294 and miR-721 inhibition increased NOS2 expression and NO production, while reducing *L*. *amazonensis* infectivity. Thus, miR-294 and miR-721 can be considered a possible targets for development of drugs against *L*. *amazonensis* [19]. The expression of miR-294-3p and miR-721 are reduced in arginase*-*deficient *L*. *amazonensis*-infected macrophages compared with wild-type *L*. *amazonensis*-infected macrophages [19]. Thus, NOS2 was up-regulated in the arginase*-*deficient *L*. *amazonensis*-infected macrophages, promoting NO production while reducing infectivity in infected macrophages. let-7e overexpression reduces NOS2 expression, whereas let-7e inhibition enhances the NOS2 expression and NO generation in *L*. *amazonensis*-infected macrophages [45].

## 6. Macrophage-related microRNAs influence the cytokine and chemokine production

Infection with R-LD leads to aggressive infection accompanied by higher production of IL-10 and TGF-β compared with S-LD infection. R-LD infection results in the NF-κB activation via ligation with TLR2/TLR6, leading to up-regulation of miR-466i in the initial hours of infection, whereas inducing high amounts of IL-10 in late hours [17]. Indeed, miR-466i may competitively interact with the IL-10 3′ UTR AU-rich sequences, which act as a binding site for an RNA-binding protein, tristetraprolin, mediating quick IL-10 mRNA degradation. Thus, miR-446i may compete out tristetrapolin from IL-103′ AU-rich sequence and prevent IL-10 mRNA degradation resulting in increased IL-10 expression in macrophages [46]. IL-10 up-regulates multidrug-resistant protein-1 (MDR-1) transporter, which effluxes antimonials from R-LD-infected cells. R-LD induces IL-10/MDR1 to expel antimonial drugs and confer a survival benefit to R-LD parasites [17]. As TLRs perform a key role in the cytokine and chemokine production by *Leishmania*-infected macrophages [47], miRNAs can directly influence cytokine and chemokine generation by targeting their genes or indirectly via the TLR-mediated signaling pathways. As most TLRs recruit general adaptor protein MyD88 to activate NF-κB, infection of MyD88-deficient macrophages, as compared to wild-type macrophages, with R-LD leads to a remarkable greater amastigote numbers accompanied by elevated IL-10/IL-12 ratio.

Unlike S-LD, R-LD infection up-regulates miR-466i that targets MyD88 mRNA, leading to its down-regulation. In S-LD-infected macrophages, HuR overexpression suppresses the pathogen-induced anti-inflammatory immune response, thereby suppressing the infection process [17]. R-LD-induced PP2A triggers anti-inflammatory responses that help parasite persistence. Thus, S-LD infection heightens HuR/PP2A ratio, causing proinflammatory macrophage responses, limiting parasite expansion [17].

The expression of CCR2, CCL5, and CXCL10 mRNAs was remarkably inhibited upon *L*. *major* infection of human macrophages [48]. Expression of let-7a, miR-25, miR-26a, miR-140, miR-146a, and miR-155, miR-23b, and miR-132 is inversely related with the CCL2, CCL5, CXCL10, CXCL11, and CXCL12 expression during the first day of infection with *L*. *major* in human macrophages [49].

In *L*. *amazonensis*-infected BALB/c macrophages, suppression of miR-30e-5p, miR-181c-5p, miR-294-3p, and miR-302d-3p decreased the parasite load. Inhibition of miR-294-3p enhanced the expression of TNF-α and MCP-1/CCL2 mRNA; inhibition of miR-30e and miR-302d promoted the RANTES/CCL5 expression and reduced the parasite infectivity [18]. miR-181c-5p inhibition reduced MCP-1 expression. RANTES/CCL5 are associated with neutrophil, monocyte, and lymphocyte recruitment to the inflammatory sites. miR-294-3p and miR-302d-3p inhibition increased TNF-α and MCP-1 expression in *L*. *amazonensis*-infected macrophages [18].

SOCS4, a suppressor of JAK-STAT-related signaling, was predicted as a target of let7a, a miRNA that is overexpressed in *L*. *donovani*-infected DCs and macrophages but is suppressed in *L*. *major*-infected cells [21]. As let-7 family miRNAs target the proinflammatory genes, their reduced expression leads to greater expression of proinflammatory cytokines in the *L*. *major*-infected cells compared to *L*. *donovani*-infected cells [21,50]. let-7e expression requires TLR2, TLR4, and MyD88-mediated signaling in *L*. *amazonensis*-infected macrophages from C57BL/6 mice. Let-7e controls the expression of proinflammatory cytokines and chemokines—IL-1, IL-6, TNF-α and MCP-1- and NOS2 in *Leishmania*-infected macrophages by targeting TLR, adaptors, and transcription factors [45].

## 7. Macrophage-related microRNAs influence CD4^+^ T-cell polarization

IL-10-induced immunosuppression plays a prominent role in PKDL development [8]. The *L*. *donovani* isolates from VL patients induce more IL-10 in macrophages than the *L*. *donovani* isolates from PKDL patients perhaps due to differential expression of miRNAs [26]. The miR-93-5p inhibition in the *L*. *donovani-*infected human macrophages promotes IL-12 production while reducing IL-10 expression [26]. miR-146, miR-155, miR-221, and miR-324 overexpression down-regulates the IFN-γR signaling in PKDL patients [26]. Bioinformatics analyses predicted that miR-29a and miR-29b would target transcription factor T-bet suppressing protective Th1 response in VL. miR-135 and miR-126 can suppress Th2 response by targeting GATA3 [24]. miR-21 and miR-590-5b can target IL-12, while miR-98 and let-7a target IL-10 suppressing Th1 and Th2-related responses, respectively [24].

miR-340 expression was down-regulated, while IL-10 and TGF-β1 were up-regulated in *L*. *major*-infected macrophages. Accordingly, a miR-340 mimic down-regulated both TGF-β1 and IL-10 and the combination of miR-340 and miR-27a exerted more impacts on target genes [51]. Overexpression of miR-340 and miR-27a reduced macrophage infectivity suggesting their plausible therapeutic utility in cutaneous leishmaniasis [51]. Alteration of miR-3473f and miR-8113 in *L*. *donovani*-infected macrophages can affect Th1/Th2 cell polarization mainly via influencing IL-12 [22]. Up-regulation of miR-6973a prevents IL-12 production and shift protective Th1 type response to Th2 type, promoting parasite survival [22]. miR-155 performs a dual role in leishmaniasis by promoting parasite persistence through prevention of apoptosis of infected macrophages (37) and VL resolution by targeting SHIP-1 and SOCS1 to enhance IFN-γ secretion from CD4^+^ Th1 responses [52]. In *L*. *donovani-*infected miR155-deficient mice, SHIP-1 and SOCS1 expression in liver and spleen was increased but initially accompanied by IFN-γ production but reduced IL-4 and IL-10 production at a later time point [52].

## 8. Macrophage-related microRNAs influence the macrophages apoptosis

Apoptosis of the *Leishmania-*infected macrophage is delayed promoting survival and immune escape of the parasite [53–55]. Therefore, targeting the apoptosis-regulating miRNAs in *Leishmania*-infected macrophages may have potential to control the infection. Indeed, miR-15a expression decreases while miR-155 increases in mouse macrophages after *L*. *major* infection [43]. miR-155 prevents macrophage apoptosis by targeting caspase 3, caspase 10, and APAF1 [56–59]. miR-15a targets antiapoptotic genes such as Bcl2 [60]. miR-15a mimics and miR-155 inhibitor increased apoptosis of *L*. *major-*infected macrophages. In *L*. *major*-infected Balb/c mice, application of miR-155 inhibitor plus a miR-15a mimic reduced parasite load and decreased the lesion sizes within 6 weeks of infection. *L*. *infantum* infection in macrophages results in the miR-155 up-regulation—greater the infectivity, higher the expression of miR-155 [20]. In fact, the *L*. *guyanensis*-expressed *Leishmania* RNA virus 1 (LRV1) increases macrophage miR-155 expression in a TLR3-dependent manner to prolong the survival of macrophage and the parasite [61]. miR-155 inhibits apoptosis by targeting Fas-associated death domain-containing protein (FADD), caspase 3, and caspase 7 [59]. miR-24-3p expression was also up-regulated in *L*. *major*-infected macrophages quickly after the infection [62]. Bioinformatics analyses indicated that miR-24-3p exerts antiapoptotic impacts by repressing the apoptotic genes such as caspases 3 and 7. miR-24-3p inhibitors may thus have the therapeutic potential against *L*. *major* infection [62].

miR-3473f, miR-763, and miR-8113 were associated with negative regulation of apoptotic process in *L*. *donovani*-infected macrophages [22]. let-7a was up-regulated in the *L*. *donovani*-infected DCs and macrophages [21]. As let-7a inhibition in macrophages increased apoptosis and necrosis, let-7a inhibition may be a potential strategy to treat leishmaniasis [63]. In the *L*. *donovani*-infected macrophages, miR-155, miR-335, miR-143, miR-221, miR-93, and let7c were associated with negative regulation of apoptosis [26]. Inhibition of antiapoptosis miRNAs can be proposed as a new strategy for treating leishmaniasis [43].

## 9. Macrophage-related microRNAs influence the macrophages autophagy

Macrophage autophagy can eliminate intracellular microorganisms, which use strategies to prevent autophagy. While miR-3473f has been implicated in the prevention of autophagy [22,31], miR-30a-3p is up-regulated early after *L*. *donovani* infection preventing macrophage autophagy but promoting parasite survival [64]. miR-30a-3p targets BECN1—an essential autophagy-promoting protein—reducing the *L*. *donovani* survival within the macrophages [64]. Likewise, miR-101c, miR-129-5p, and miR-210-5p can influence autophagy via targeting the autophagy-related 4 (ATG4), specificity protein 1 (SP1) (a CTSE expression regulator), and BNIP3, respectively. Transfection of *L*. *major*-infected MDM with miR-101c and miR-129-5p mimics, or with a miR-210-5p inhibitor decreases infection load [65]. *L*. *major*-infected macrophages’ autophagy was correlated with miR-101c and miR-129-5p overexpression and the low miR-210 expression [65]. Autophagy targeting in *Leishmania*-infected macrophages can be considered as a plausible antileishmanial therapy.

## 10. Macrophage-related microRNAs influence the phagosome maturation and drug resistance

Following internalization, the pathogen-containing phagosome undergoes sequential maturation including the early-, intermediate-, and late phagosome formation. The late phagosome is fused with lysosomes to form phagolysosomes, resulting in degradation of phagosomal contents. The maturation processes are regulated by several factors, the incredibly sequential shift from the early endosomal Rab5 to the late endosomal Rab7 [66]. Following internalization, *L*. *donovani* secretes gp63 that inactivates c-Jun to down-regulate the miR-494 expression, thereby leads to the Rab5a overexpression in the infected cells [67]. Subsequently, *L*. *donovani* recruits and preserves Rab5a on its parasitophorous vacuoles to persist in the early endosomes and suppresses their merging with lysosomes. miR-494 negatively regulates Rab5a expression limiting parasite survival [67]. miRNAs play an important role in drug resistance in infectious and noninfectious diseases [68,69]. *Leishmania* can induce ABC transporters overexpression in macrophages by down-regulating miR-763, miR-1264, and miR-3473f that help the drug efflux from the *Leishmania*-infected cells [22].

## 11. Regulation of miRNA expression by inflammatory cytokines and mediators

There is a reciprocal regulatory relationship between miRNAs and inflammatory mediators/cytokines. For example, miR-187 has been identified as an IL-10-associated miRNA playing a role in IL-10-related suppression of TNF-α, IL-6, and the IL-12p40 by primary human monocytes following TLR4 stimulation [70]. IL-10 attenuates inflammatory responses by suppressing the expression of miR-155 in LPS-stimulated macrophages. miR-155 promotes the expression of proinflammatory cytokines such as TNF-α and inhibits the expression of anti-inflammatory elements as SOCS1. IL-10 had no effect on miR-155 gene transcription or nuclear export of pre-miR-155, but it destabilized both pri-miR-155 and pre-miR-155 transcripts, as well as interfered with miR-155 maturation [71]. The inhibitory impact of IL-10 on miR-155 expression was mediated via STAT3 and SHIP1, as IL-10-mediated inhibition of miR-155 expression was hampered in SHIP1-deficient macrophages but a SHIP1 activator restored this inhibition [71] and a STAT3 inhibitor reduced IL-10-mediated inhibition of miR-155. IL-10-induced SHIP1 prevents AKT-related signaling and lowers miR-155 expression [71]. IL-10-mediated miR-155 down-regulation led to the overexpression of the miR-155 target, SHIP1 [72]. IL-10 had no influence on miR-21 or miR-146a expression but it induced miR-146b in LPS-treated human and mouse macrophages, as IL-10-deficient macrophages had less miR-146b expression [73]. Once expressed, miR-146b targets TLR4 mRNA as well as critical adaptor/signaling factors such as MyD88, IRAK1, and TRAF6 leading to anti-inflammatory effects [73]. As an IL-10-dependent miRNA, miR-146b thus exerts anti-inflammatory activity by targeting of TLR4-related signaling components in macrophages [73]. In LPS-stimulated mouse macrophages, IL-10 suppresses the expression of miR-147, while increasing the expression of miR-455. The inhibition of miR-147 by IL-10 implies that miR-147 may have a proinflammatory role in TLR-activated macrophages [74].

IL-4 and IL-13, the cytokines secreted from Th2 cells, up-regulate miR-142-5p and down-regulate miR-130a-3p that play a key role in M2 polarization of macrophages [75]. The miR-142-5p overexpression by IL-4 suppresses SOCS1, a negative regulator of STAT-6, and thereby prolongs the life of phospho-STAT-6. STAT6-induced SOCS1 expression thus provides a negative feedback loop. A robust M2 polarization needs the involvement of PPAR, which is down-regulated in M1 macrophages but up-regulated in M2 macrophages [75]. IL-4 promotes histone deacetylation at the promoter of miR-130a-3p to reduce its expression and eliminate the suppressive effect of miR-130a-3p on the PPAR expression [76]. LPS represses PPAR expression by up-regulating miR-27b in M1 macrophages [76]. Differently expressed miRNAs in polarized macrophages may thus play a role in the maintenance of their activation [76].

The activation of classical M1 macrophages requires priming with IFN-γ followed by TLR stimulation. The IFN-γ priming causes macrophages to produce more proinflammatory, but less anti-inflammatory, cytokines and to boost their microbicidal and tumoricidal activities. IFN-γ reduces miR-3473b expression. miR-3473b promotes Akt/glycogen synthase kinase 3 signaling and IL-10 generation by directly targeting phosphatase and tensin homolog (PTEN) to decrease macrophage activation and inflammatory response [77]. The IFN-γ/miR-3473b/PTEN axis suggests a positive feedback loop whereby IFN-γ targets miR-3473b and allows de-repression of PTEN that inhibits Akt signaling and IL-10 production to eventuate in the activation of IFN-γ-primed macrophages [77].

TGF-β, a *Leishmania*-induced cytokine that is involved in Treg cells differentiation, regulates miRNA expression. TGF-β augments the expression of miR-29b that promotes the expression of Cyclooxygenase-2—catalyzing arachidonic acid conversion to prostaglandins (PG), reduces the bactericidal activity of macrophages [78]. Prostaglandin E2 (PGE2)-mediated macrophage polarization is regulated by miR-21 [79], as PGE2 down-regulates miR-21 that influences the M2 macrophage-associated genes expression [80]. Type-I IFNs down-regulates miR-145 expression. miR-145 targets the IL-10 gene silencer histone deacetylase 11 (HDAC11) leading to higher IL-10 expression in macrophages [81]. As Type-I IFNs suppress miR-145, the type I IFN/miR-145/HDAC11 axis thus suppresses IL-10 production by relieving HDAC11 from the miR-145 targeting [81].

### 12. Limitations in the studies on miR-regulation of *Leishmania* infection and antileishmanial immune response

Herein, we have extensively reviewed the available reports on the roles of miRs at the interface of *Leishmania*-macrophage interaction. Although efforts are made to align these reports to develop a generalized framework of *Leishmania*-modulating miRs expression and miRs-regulating antileishmanial immune response, it is limited by the lack of mechanistic details, incomparability between the model systems used, contradictions between the reported effects, *Leishmania* species-dependent variations in the miRs and their effects, heterogeneity of macrophages and dynamicity in the regulation of miRs expression, and effects on *Leishmania* infection. Understanding the evolution of the regulation of miRs expression and activities will require extensive coordinated interdisciplinary investigations using 1 system of host macrophage and 1 *Leishmania* species at a time.

## 13. Conclusions

**Fig 5:**
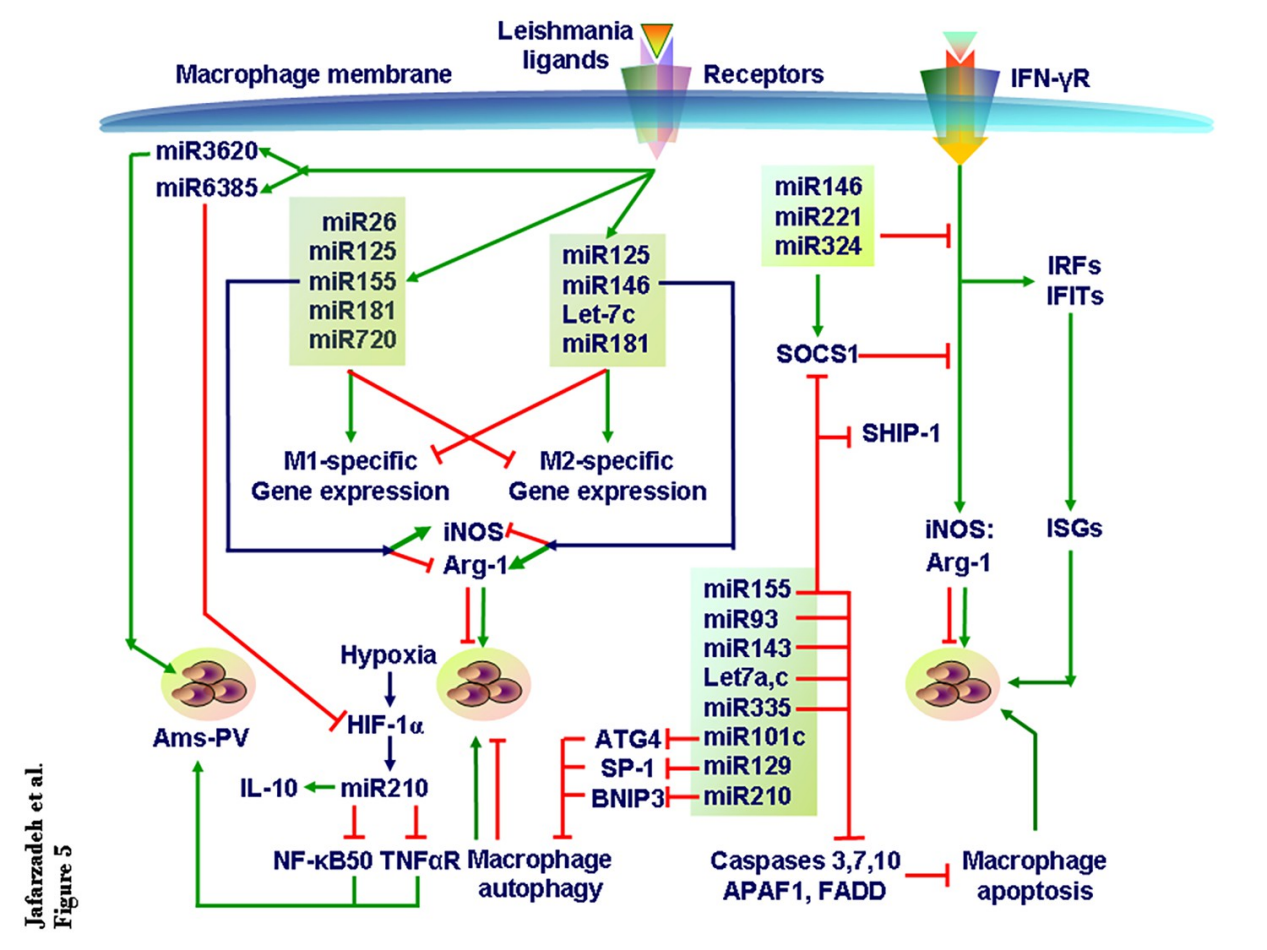
miRNA regulation of *Leishmania* infection. TGF-β, and perhaps other cytokines too, regulate miRNA expression in macrophages. As *Leishmania* infection renders the intracellular environment hypoxic, the HIF-1α induces miR210 to enhance IL-10 production but reduce NF-κB activation and TNF-αR expression to enhance parasite load in the parasitophorous vacuoles in macrophages. Various miRNAs expressed during *Leishmania* infection modulate cellular processes including cytokine signaling that affects the outcome of *Leishmania* infection. Similarly, 2 counteractive sets of miRs reciprocally regulate M1 and M2 macrophage subsets differentiation that affects parasite growth. The other miRs regulate macrophage Autophagy and apoptosis and IFN-γR signaling to alter intracellular amastigote numbers. The available literature is too diverse in terms of *Leishmania* species, macrophage populations, experimental models, and assays to derive a coherent and integrative view of miRs role in *Leishmania* infection. Therefore, only a fraction of the literature is used to develop the current perspective. HIF-1α, hypoxia-inducible factor-1α; miRNA, microRNA.

miRNAs play pivotal roles in the macrophage-mediated antileishmanial responses such as NOS2 and cytokine expression (Fig 5). In turn, the parasite and the cytokines regulate the expression of multiple miRNAs. Two different mechanisms operate simultaneously: An individual miRNA may target multiple genes and several miRNAs may target 1 particular gene. Following this bidirectional miRNA-based immunoregulatory framework may identify the cocktail of the miRNA mimics or miRNA inhibitors for an efficient antileishmanial therapy.
